# Effects of childhood experiences of parental attitude, depressive rumination, and sleep disturbances on adulthood depressive symptoms

**DOI:** 10.1002/pcn5.220

**Published:** 2024-06-24

**Authors:** Shinichi Akiyama, Miki Ono, Yoshitaka Ishii, Masayuki Kikkawa, Shunichiro Ito, Mina Honyashiki, Yu Tamada, Hironori Takeuchi, Takeshi Inoue, Jiro Masuya

**Affiliations:** ^1^ Department of Psychiatry Tokyo Medical University Tokyo Japan; ^2^ Department of Pharmacy Tokyo Medical University Tokyo Japan; ^3^ Department of Psychiatry Tokyo Medical University Hachioji Medical Center Tokyo Japan

**Keywords:** depressive rumination, depressive symptoms, parental attitudes, sleep disturbance, structural equation modeling

## Abstract

**Aim:**

Various factors are thought to be involved in the development of depression, but the mechanisms are not yet clear. Although several reports have demonstrated that parental attitude experienced in childhood, depressive rumination, and sleep disturbances each influence depressive symptoms, and the association between two of these four variables, to our knowledge, no reports to date have investigated the association among the four variables.

**Methods:**

A questionnaire survey was administered to 576 adults who agreed to participate in this study between April 2017 and April 2018. Questionnaires assessed parental attitudes experienced in childhood, depressive rumination, sleep disturbances, and depressive symptoms in adulthood. The associations among the four variables were tested by structural equation modeling.

**Results:**

Regarding the direct effects, the parental attitude of “care” had a negative influence on depressive rumination and depressive symptoms, whereas “overprotection” had a positive influence on depressive rumination. Depressive rumination had a positive influence on sleep disturbance and depressive symptoms, whereas sleep disturbances had a positive influence on depressive symptoms. Regarding indirect effects, depressive rumination mediated the association between parental attitudes and sleep disturbances or depressive symptoms. Furthermore, sleep disturbances mediated the association between depressive rumination and depressive symptoms. Care and overprotection showed opposite effects. The goodness of fit of this model was high.

**Conclusion:**

The results of this study demonstrated that there were associations among the four variables. Clinical assessment and intervention of depressive rumination and sleep disturbances that are closely associated with previous parental attitudes may lead to an improvement of depressive symptoms.

## Graphical abstract

Results of the structural equation model with the parental attitude of “care” experienced in childhood as the latent variable, and depressive rumination (Ruminative Responses Scale), sleep disturbance (Pittsburgh Sleep Quality Index), and depression evaluated on the Patient Health Questionnaire‐9 as the observed variables.

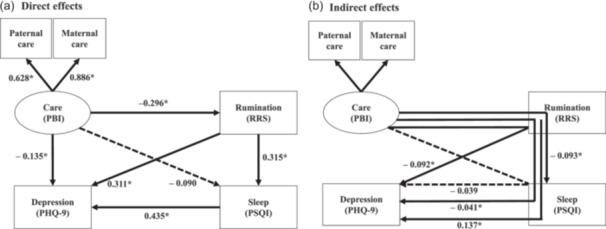

## INTRODUCTION

Depression results in a variety of symptoms, including depressed mood, a substantial decrease in interest or pleasure, changes in weight, changes in sleep patterns, agitation or inhibition of psychomotor ability, fatigue or decreased energy, worthlessness or inappropriate guilt, the decreased ability to think or concentrate, and suicidal ideas or suicide attempts.^1^ Its lifetime prevalence rate varies from 3% to 20.6%, and it is a common psychiatric disorder.^2^, ^3^ Approximately 280 million people in the world have depression, which is the leading cause of disability and a major disease burden.^4^, ^5^ The etiology of depression has been the subject of numerous studies, and includes innate individual factors, such as genetic risk factors and temperamental factors.^6^ Other etiological factors are also involved, including environmental factors, such as adverse psychosocial experiences in childhood and adulthood (e.g., inappropriate parenting and stressful life events), depressive rumination, sleep disturbances, childhood anxiety and conduct disorders, and substance use disorders.^7^, ^8^, ^9^, ^10^ These factors are believed to complexly influence the onset of depression, but how these factors influence each other remains unclear.

Among the environmental factors influencing depression, the nurturing environment is a factor that has received much attention in recent years. Several previous studies have shown that inappropriate parental nurturing, such as low care (i.e., emotional coldness or neglect) and high overprotection (i.e., interference or control of independent behavior) by parents toward their children, have a negative impact on depression and depressive symptoms in adulthood.^7^, ^11^, ^12^ A cross‐sectional study of medical students found that the experience of adequate parental nurturing in childhood attenuated depressive symptoms, whereas inappropriate parental nurturing (particularly high overprotection) exacerbated depressive symptoms.^13^ We have also found in previous studies that inappropriate parental nurturing (particularly low care and high overprotection) exacerbates depressive symptoms mediated by high neuroticism.^14^

“Repetitive thought” is the process of thinking attentively, repetitively, or frequently about one's self and one's world, and one type of repetitive thought is “depressive rumination.”^15^ Depressive rumination is defined as repeated passive thoughts about one's depression and its causes and results.^16^ Depressed patients are known to have a higher frequency of depressive rumination than healthy individuals.^17^ Furthermore, depressive rumination predicts or exacerbates depression.^8^, ^18^ Our previous studies have shown that depressive rumination in adulthood mediates the association between childhood abuse, trait anxiety, and depressive symptoms.^19^

Sleep disturbances are a symptom of depression, and treating sleep disturbances in patients with depression reportedly improves depressive symptoms.^20^, ^21^ On the other hand, sleep disturbances are also a causal factor of the onset of depression; two meta‐analyses demonstrated that patients with insomnia were more than twice as likely to develop depression.^9^, ^10^ Furthermore, our previous studies have shown that sleep disturbances interact with resilience to influence depressive symptoms.^22^ Thus, a bidirectional association has been shown to exist between sleep disturbances and depression.

Regarding “parental attitudes” and “sleep disturbances,” there are reports of inappropriate parenting resulting in sleep disturbances in adolescents and adults.^23^, ^24^, ^25^ Regarding “parental attitudes” and “depressive rumination,” college students who felt that they had inadequate parental nurturing reported a higher frequency of rumination.^26^ Inadequate parenting has also been reported to increase the frequency of rumination in studies on children.^27^, ^28^ There have been many reports to date on “depressive rumination” and “sleep disturbances.” In a longitudinal study of 42 college students with depression, Pillai et al. reported that sleep latency was prolonged in students with a higher frequency of depressive rumination.^29^ A cross‐sectional study in adults aged 20 to 80 years also showed an association between depressive rumination, sleep disturbances, and sleep‐associated impairments.^30^ A meta‐analysis of 55 studies showed that a higher frequency of depressive rumination was associated with longer sleep onset latency, shorter total sleep duration, and poorer sleep quality.^31^ Therefore, we hypothesized that depressive rumination mediates the association between parental attitudes and sleep disturbances.

Although the above‐mentioned associations between two variables have been reported, such as “each variable and depression,” “parental attitudes and sleep disturbances,” “parental attitudes and depressive rumination,” and “depressive rumination and sleep disturbances,” there are no reports to our knowledge on the associations among all four of the variables, including mediating effects. Therefore, in this study we hypothesized that parental attitudes influence depressive rumination, which in turn influences depressive symptoms through its impact on sleep disturbances. Parental attitudes, depressive rumination, sleep disturbances, and depressive symptoms were assessed in adult volunteers by a paper‐based questionnaire survey, and the associations among these variables were tested by structural equation modeling (SEM).

## METHODS

### Participants

Self‐administered questionnaires were distributed to 1237 adult volunteers between April 2017 and April 2018. The study was part of a larger study.^19^, ^22^ Of these, 576 adult volunteers who provided valid responses and written informed consent were included. The inclusion criterion was being 20 years old or older. The exclusion criteria were having a severe physical illness or organic mental illness. The participants were informed that participation in the study was voluntary, and that there would be no disadvantage for not participating or withdrawing from the study, and that personal information would be kept confidential. The institutional review board of Tokyo Medical University (study approval no.: SH3502) approved this study in accordance with the Declaration of Helsinki (amended in 2013).

### Evaluation items

The variables evaluated included “demographic information,” “depressive symptoms,” “experiences of parental attitudes in childhood,” “depressive rumination,” and “sleep disturbances.” The following questionnaires were used to evaluate the four variables other than demographic information.

### Questionnaires

#### Patient Health Questionnaire‐9

The Patient Health Questionnaire‐9 (PHQ‐9) is a rating scale that subjectively evaluates the severity of depressive symptoms experienced in the previous 2 weeks, and includes nine items.^32^ The Japanese version of PHQ‐9 was translated and validated by Muramatsu et al.^33^ Total scores (0–27) were used for analysis in this study.

#### Parental Bonding Instrument

The Parental Bonding Instrument (PBI) is a retrospective rating scale of parental attitudes from the child's perspective up to the age of 16 years, using 25 questions (12 care and 13 overprotection items).^34^ Higher scores on care indicate that the children had received appropriate nurturing from their parents (closeness, affection, empathy, emotional warmth, etc.) and higher scores on overprotection indicate that the children had received overprotection from their parents (control, intrusion, excessive contact, infantilization, etc.). Some of the items are as follows: “Seemed emotionally cold to me,” “Did not want me to grow up,” “Invaded my privacy,” “Did not seem to understand what I needed or wanted,” and “Did not praise me.” These examples indicate that low parental care and high parental overprotection evaluated by the PBI are adverse experiences. In the authors’ unpublished data, the care score on the PBI showed a significant moderate negative correlation (maternal: *r* = –0.588; paternal: *r* = –0.613) with the total score for the Child Abuse and Trauma Scale (CATS), which was used as an index of childhood abuse. The overprotection score on the PBI showed a significant moderate positive correlation (maternal: *r* = 0.504; paternal: *r* = 0.629) with the CATS total score. The Japanese version of the PBI was translated and validated by Kitamura and Suzuki.^35^ In this study, care (0–36 points) and overprotection (0–39 points) for both the father and the mother were used for the analysis.

#### Ruminative Responses Scale

The Ruminative Responses Scale (RRS) is a rating scale that subjectively assesses the frequency of depressive rumination using 22 questions.^36^ Higher scores indicate a higher frequency of depressive rumination. The Japanese version of the RRS was translated and validated by Hasegawa.^37^ Total scores (22–88) were used for analysis in this study.

#### Pittsburgh Sleep Quality Index

The Pittsburgh Sleep Quality Index (PSQI) is a rating scale that subjectively evaluates sleep disturbances during the previous month using 19 questions to rate seven subscales (C1: Sleep Quality; C2: Sleep Latency; C3: Sleep Duration; C4: Habitual Sleep Efficiency; C5: Sleep Disturbance; C6: Use of Sleeping Medication; and C7: Daytime Dysfunction).^38^ The Japanese version of the PSQI was translated and validated by Doi et al.^39^, ^40^ Global scores (total score of seven subscales, 0–21) were used for analysis in this study. A higher global score indicates more severe sleep disturbances, and 6 points or greater is considered to indicate a sleep disturbance.

### Statistical analysis

In this study, univariate (*t*‐test and Pearson correlation coefficient) and multivariate (multiple regression analysis [forced entry method]) analyses of associations or correlations between demographic data and questionnaire scores were conducted using SPSS 28 software (IBM). The effects of “parental attitude” (PBI, care and overprotection) on “depressive symptoms in adulthood” (PHQ‐9) were hypothesized to be mediated by “depressive rumination” (RRS) and “sleep disturbances” (PSQI). SEM was performed using a model in which the latent variable “care” was created from the observed variables of paternal care and maternal care, and the latent variable “overprotection” was created from the observed variables of paternal overprotection and maternal overprotection. The hypothesized model that care or overprotection in these models affects PHQ‐9 via RRS and PSQI was tested. The root‐mean‐square error of approximation (RMSEA) and comparative fit index (CFI) were used as indexes of goodness of fit. An RMSEA of <0.05, and a CFI of >0.97 were considered to indicate a good fit; and an RMSEA of <0.08, and a CFI of >0.95 were considered to indicate an acceptable fit.^41^ The SEM was performed using Mplus8.5 software (Muthén & Muthén). A *p*‐value of <0.05 was considered to indicate a statistically significant difference.

## RESULTS

### Association of demographic characteristics, and correlation of PBI, RRS, and PSQI scores with PHQ‐9 scores (Table 1)

**Table 1:** Associations and correlations of PHQ‐9 scores with demographic characteristics, PBI, RRS, and PSQI scores in 576 adult volunteers.

Characteristic or measure	Value (number or mean ± SD)	Correlation with PHQ‐9 score (*r*) or effect on PHQ‐9 score (mean ± SD, *t*‐test)
PHQ‐9 total score	4.1 ± 4.3	−
Age (years)	41.6 ± 12.0	*r* = −0.032, *p* = 0.448 (n.s.)
Sex (men : women)	249 : 327	Men: 3.5 ± 4.1 Women: 4.5 ± 4.3, *p* = 0.005 (*t*‐test) **
Marital status (married : unmarried)	377 : 194	Married: 3.5 ± 3.9 Unmarried: 5.2 ± 4.7, *p* < 0.001 (*t*‐test) ***
Living alone (no : yes)	462 : 114	No: 3.8 ± 4.1 Yes: 5.1 ± 4.7, *p* = 0.004 (*t*‐test)**
Education (years)	14.6 ± 1.8	*r* = −0.073, *p* = 0.078 (n.s.)
Past history of psychiatric disease (yes : no)	68 : 508	Yes: 6.7 ± 5.3 No: 3.7 ± 4.0, *p* < 0.001 (*t*‐test)***
Current psychiatric disease (yes : no)	23 : 544	Yes: 7.8 ± 5.0 No: 3.9 ± 4.2, *p* < 0.001 (*t*‐test)***
Family history of psychiatric disease (yes : no)	58 : 463	Yes: 4.5 ± 4.2 No: 4.0 ± 4.3, *p* = 0.362 (*t*‐test) (n.s.)
RRS score	35.2 ± 11.4	*r* = 0.499, *p* < 0.001***
PBI score		
Paternal care	23.5 ± 8.2	*r* = −0.179, *p* < 0.001***
Maternal care	28.0 ± 7.0	*r* = −0.273, *p* < 0.001***
Paternal overprotection	9.7 ± 7.0	*r* = 0.209, *p* < 0.001***
Maternal overprotection	9.7 ± 7.0	*r* = 0.238, *p* < 0.001***
PSQI global score	5.8 ± 3.5	*r* = 0.566, *p* < 0.001***

*Note*: Data are presented as the mean ± standard deviation (SD) or number. *r* = Pearson correlation coefficient.

Abbreviations: n.s., not significant; PBI, Parental Bonding Instrument; PHQ‐9, Patient Health Questionnaire‐9; PSQI, Pittsburgh Sleep Quality Index; RRS, Ruminative Responses Scale.

** *p* < 0.01.

*** *p* < 0.001.

Data on demographic characteristics, parental attitudes (PBI, care and overprotection), depressive rumination (RRS), sleep disturbances (PSQI), and depressive symptoms (PHQ‐9) of the 576 adult volunteers are presented in Table 1. Subjects who were female, unmarried, living alone, with a past history of psychiatric disease, and with current psychiatric disease had significantly higher PHQ‐9 scores. A family history of psychiatric disease had no significant association with PHQ‐9 score. RRS, paternal overprotection, maternal overprotection, and PSQI scores showed significantly positive correlations with PHQ‐9 scores, whereas paternal care and maternal care showed significantly negative correlations with PHQ‐9 scores. Age and years of education showed no significant correlation with PHQ‐9 scores.

Although not shown in Table 1, the PBI care score showed a significant negative correlation with the RRS score (maternal: *r* = –0.255, *p* < 0.001; paternal: *r* = –0.195, *p* < 0.001), and the PBI overprotection score showed a significant positive correlation with the RRS score (maternal: *r* = 0.293, *p* < 0.001; paternal: *r* = 0.197, *p* < 0.001). Both care and overprotection scores were correlated more strongly with the RRS score for mothers than that for fathers.

### Results of multiple regression analysis with PHQ‐9 as a dependent variable (Table 2)

**Table 2:** Results of multiple regression analysis with PHQ‐9 as a dependent variable (forced entry method).

Independent variable	Standardized partial regression coefficient (β)	*p*‐value	VIF
Age	–0.007	0.872	1.424
Sex (women)	0.056	0.132	1.154
Marital status (married)	0.037	0.428	1.830
Living alone	–0.087	0.057	1.726
Education (years)	–0.008	0.857	1.557
Past history of psychiatric disease	0.080	0.058	1.462
Current psychiatric disease	0.006	0.889	1.413
Family history of psychiatric disease	–0.030	0.406	1.089
RRS score	0.295	<0.001	1.435
PBI score			
Paternal care	0.067	0.179	2.052
Maternal care	–0.181	0.001	2.394
Paternal overprotection	0.070	0.179	2.270
Maternal overprotection	–0.091	0.093	2.437
PSQI global score	0.394	<0.001	1.311
Adjusted *R* ^2^ = 0.46, *F* = 26.65, *p* < 0.001			

Abbreviations: PBI, Parental Bonding Instrument; PSQI, Pittsburgh Sleep Quality Index; PHQ‐9, Patient Health Questionnaire‐9; RRS, Ruminative Responses Scale; VIF, variance inflation factor.

Table 2 shows the results of multiple regression analysis using PHQ‐9 as the dependent variable. Of the 14 independent variables, RRS, maternal care, and PSQI score were the three independent variables that significantly predicted the PHQ‐9 score. The adjusted *R* ^2^ was 0.46. Multicollinearity was ruled out.

### SEM results for the parental‐care–RRS–PSQI–PHQ‐9 hypothetical model (Figure 1)

**Figure 1:**
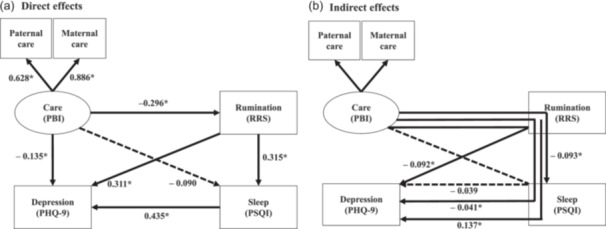
Results of the structural equation model with the parental attitude of “care” experienced in childhood as the latent variable, and depressive rumination (Ruminative Responses Scale [RRS]), sleep disturbance (Pittsburgh Sleep Quality Index [PSQI]), and depression evaluated on the Patient Health Questionnaire‐9 (PHQ‐9) as the observed variables. The latent variable is shown as an oval, and the observed variables are shown as rectangles. The arrows with solid lines represent the statistically significant paths, and those with broken lines represent the nonsignificant paths. (a) Direct effects and (b) indirect effects between the variables are shown. The numbers show the standardized path coefficients. *p < 0.001. PBI, Parental Bonding Instrument.

An RMSEA of 0.072 and a CFI of 0.988 indicated that the model fit was acceptable. The standardized coefficients (β) from care to paternal care and maternal care were 0.628 and 0.886, respectively; the latter being larger. Results for the direct effects are shown in Figure 1a, where care had a significant negative effect on PHQ‐9 (β = –0.135, *p* < 0.001) and RRS (β = –0.296, *p* < 0.001), but a nonsignificant effect on PSQI (β = –0.090, *p* = 0.147). RRS had a significant positive effect on PSQI (β = 0.315, *p* < 0.001) and PHQ‐9 (β = 0.311, *p* < 0.001). PSQI had a significant positive effect on PHQ‐9 (β = 0.435, *p* < 0.001).

Results for the indirect effects are shown in Figure 1b, in which care had a significant negative impact on PHQ‐9 (β = –0.041, *p* < 0.001) via RRS and PSQI. Care also had a significant negative impact on PHQ‐9 (β = –0.092, *p* < 0.001) and PSQI (β = –0.093, *p* < 0.001) via RRS. Care did not affect PHQ‐9 significantly (β = –0.039, *p* = 0.148) via PSQI. RRS had a significant positive effect on PHQ‐9 (β = 0.137, *p* < 0.001) via PSQI. In other words, in the present model, all pathways that significantly affected PHQ‐9 were via the RRS. The coefficient of determination *R* ^2^ for PHQ‐9 was 0.443, and this model explained 44.3% of the variability of depressive symptoms in adult volunteers.

### SEM results for the parental‐overprotection–RRS–PSQI–PHQ‐9 hypothetical model (Figure 2)

**Figure 2:**
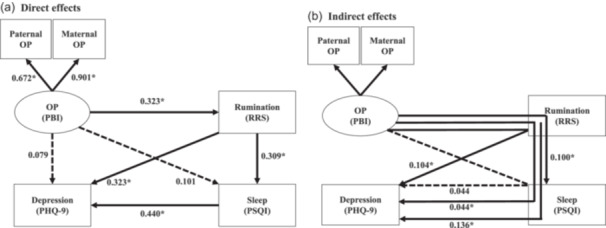
Results of the structural equation model with the parental attitude of “overprotection (OP)” in childhood as the latent variable, and depressive rumination (Ruminative Responses Scale [RRS]), sleep disturbance (Pittsburgh Sleep Quality Index [PSQI]), and depression evaluated on the Patient Health Questionnaire‐9 (PHQ‐9) as the observed variables. The latent variable is shown as an oval, and the observed variables are shown as rectangles. The arrows with solid lines represent the statistically significant paths, and those with broken lines represent the nonsignificant paths. (a) Direct effects and (b) indirect effects between the variables are shown. The numbers show the standardized path coefficients. *p < 0.001. PBI, Parental Bonding Instrument.

An RMSEA of 0.041 and a CFI of 0.996 indicated that the model fit was good. The standardized coefficient β from overprotection to paternal overprotection and maternal overprotection was 0.672 and 0.901, respectively; the latter being larger. Results for direct effects are shown in Figure 2a, in which overprotection showed a significant positive effect on RRS (β = 0.323, *p* < 0.001) and a nonsignificant effect on PHQ‐9 (β = 0.079, *p* = 0.067) and PSQI (β = 0.101, *p* = 0.062). RRS had a significant positive effect on PSQI (β = 0.309, *p* < 0.001) and PHQ‐9 (β = 0.323, *p* < 0.001). PSQI had a significant positive effect on PHQ‐9 (β = 0.440, *p* < 0.001).

Results for the indirect effects are shown in Figure 2b, in which overprotection had a significant positive effect on PHQ‐9 (β = 0.044, *p* < 0.001) via RRS and PSQI. Overprotection also had a significant positive effect on PHQ‐9 (β = 0.104, *p* < 0.001) and PSQI (β = 0.100, *p* < 0.001) via RRS. Overprotection did not significantly affect PHQ‐9 (β = 0.044, *p* = 0.065) via PSQI. RRS had a significant positive effect on PHQ‐9 (β = 0.136, *p* < 0.001) via PSQI. In other words, in the present model, all pathways that significantly affected PHQ‐9 were via the RRS. The coefficient of determination *R* ^2^ for PHQ‐9 was 0.432, and this model explained 43.2% of the variability of depressive symptoms in adult volunteers.

Care and overprotection showed opposite effects (Figures 1, 2). Significant direct and indirect effects were similar between care and overprotection, except for the direct effect on PHQ‐9.

### Results of logistic regression analysis and SEM using PHQ‐9 score ≥10 or PHQ‐9 score ≤4 as the dependent variable (forced entry method) (Table S1 and Figures S1, S2)

Following the criteria of a PHQ‐9 score of ≥10 indicating major depression (MD) and a PHQ‐9 score of ≤4 indicating no depression, based on a previous study on the validity of assessing depression severity using the PHQ‐9 score,^42^ 8.8% of the subjects in the present study had MD (data not shown). Logistic regression analysis was performed with the dependent variable of MD or PHQ‐9 score ≤4 (no depression), followed by SEM with MD or no depression as the dependent variable.

Logistic regression analyses using the forced entry method were conducted with nine independent variables, including age, sex, RRS score, PBI scores, and PSQI global score. Table S1 shows that RRS score, maternal care score on the PBI, and PSQI global score are factors that significantly differentiate PHQ‐9 score ≥10 (MD) from PHQ‐9 score ≤4 (no depression).

The SEM results were almost the same as the results when the PHQ‐9 total score was used as the dependent variable (Figures 1, 2). The PBI score (care and overprotection), RRS score, and PSQI global score both directly and indirectly affected the differentiation between MD and no depression (Figures S1 and S2). An RMSEA of 0.037 and a CFI of 0.997 in Figure S1 (care), and an RMSEA of 0.035 and a CFI of 0.998 in Figure S2 (overprotection) indicated that the fit was good for both models.

### Results of multiple regression analysis and SEM of total scores of the eight items of the PHQ‐9 without the sleep item (Item no. 3) as the dependent variable (forced entry method) (Table S2 and Figures S3, S4)

The global score of the PSQI differs from the sleep item of the PHQ‐9 (Item no. 3) in that the PSQI assesses sleep disturbances more comprehensively and in more detail. However, as a moderate correlation between the sleep item of the PHQ‐9 and the PSQI global score was observed (*r* = 0.57, *p* < 0.001, data not shown), the effect of the PSQI global score on the PHQ‐9 total score may be explained by the fact that these questionnaires are partly identical. To rule out this possibility, multiple regression analyses (forced entry method) and SEM were conducted using the total score of eight items of the PHQ‐9 without the sleep item (Item no. 3) as the dependent variable.

The results of the multiple regression analysis (forced entry method) showed that of the 14 independent variables, the RRS score, maternal care score on the PBI, paternal overprotection score on the PBI, PSQI global score, and past history of psychiatric disease were the five independent variables that significantly predicted the total score of the eight items of the PHQ‐9 without the sleep item (Item no. 3) (Table S2). The adjusted *R* ^2^ was 0.43, and multicollinearity was ruled out.

The results of the SEM were the same as those when the PHQ‐9 total score (nine items) was used as the dependent variable. The PBI score (care and overprotection), RRS score, and PSQI global score directly and indirectly affected the total score of the eight items of the PHQ‐9 without the sleep item (Item no. 3) (Figures S3, S4). An RMSEA of 0.076 and a CFI of 0.986 in Figure S3 (care), and an RMSEA of 0.045 and a CFI of 0.995 in Figure S4 (overprotection) indicate that the model fit was acceptable and good, respectively.

### Path analysis results for the maternal‐care‐ and paternal‐care‐RRS–PSQI–PHQ‐9 models (Figures S5, S6)

As maternal care showed a larger effect on depressive symptoms than paternal care (Tables 1 and 2), path analyses for maternal care and paternal care were conducted in separate models (Figures S5, S6). Maternal care had a significant direct negative effect (β = –0.131, *p* < 0.001) on PHQ‐9 scores, whereas paternal care had no significant direct effect on PHQ‐9 scores (β = –0.037, *p* = 0.288). In addition, paternal care (β = –0.116, *p* = 0.009) had a significant direct negative effect on sleep disturbances, whereas maternal care did not (β = –0.059, *p* = 0.174). Other results of direct and indirect effects were mostly similar to the results of SEM with parental care as a latent variable (Figure 1). Accordingly, maternal care and paternal care showed similar indirect effects on sleep disturbances and depressive symptoms through depressive rumination.

## DISCUSSION

In this study, we hypothesized that parental attitudes experienced in childhood, such as care and overprotection, influence depressive rumination, and furthermore that depressive rumination influences sleep disturbances, which in turn influence depressive symptoms. We tested this hypothesis using SEM. We found that depressive rumination mediates the association between parental attitudes and sleep disturbances or depressive symptoms. Furthermore, we found that sleep disturbances mediate the association between depressive rumination and depressive symptoms. This study is the first to report the mediating effects of “adult depressive rumination” and “adult sleep disturbances” by an integrated analysis of these four variables.

In this study, parental attitudes experienced in childhood had no significant direct effect on sleep disturbances, but the indirect effect of parental attitudes on sleep disturbances in adulthood was fully mediated by depressive rumination. Depressive rumination also had a direct exacerbating effect on sleep disturbances. Prior studies have reported that college students who received inadequate parental care have an increased frequency of depressive rumination.^26^ Furthermore, several studies have reported that enhanced rumination exacerbates sleep disturbances.^29^, ^30^, ^31^, ^43^ The mediating effect of depressive rumination shown in the present study linked the above findings each other.^26^, ^29^, ^30^, ^31^, ^43^ Moreover, our finding proposes the mechanism of how inappropriate parenting experienced in childhood causes sleep disturbances in adulthood^25^; that is, depressive rumination mediates the effect of parenting on sleep.

We further found that depressive rumination and sleep disturbances each had a direct exacerbating effect on depressive symptoms. Prior studies have reported that patients with depression have a higher frequency of depressive rumination than healthy individuals, and that depressive rumination predicts or exacerbates the onset of depression.^8^, ^17^, ^18^ It has also been reported that patients with insomnia are more than twice as likely to develop depression.^9^, ^10^ Thus, both depressive rumination and sleep disturbances have been associated with depression and depressive symptoms in adults. The clinically useful information obtained from this study is that the effect of depressive rumination on depressive symptoms is partially mediated by sleep disturbances. The reverse direction in the SEM may be possible; that is, depressive symptoms may exacerbate sleep, further leading to frequent depressive rumination. However, in the present study, depressive rumination was considered as a personality characteristic, which is relatively stable in individuals,^8^ and we assessed sleep disturbances in life during the previous month, and depressive symptoms in the previous 2 weeks. Therefore, the direction of the paths from depressive rumination to sleep disturbances and then to depressive symptoms appears plausible.

As reported in our previous study analyzing another group of patients,^44^ and similar to other previous studies,^28^, ^45^, ^46^, ^47^ the results of our present study demonstrated that maternal attitude influences depressive symptoms and depressive rumination more than paternal attitude. Considering the age of the subjects of the present study and the characteristics of the Japanese population, it is likely that the difference in the effects of maternal and paternal care and overprotection is owing to the fact that mothers are generally the primary caregivers during a person's childhood.

The results of our present study suggest that appropriate parenting in childhood reduces depressive rumination, and that the reduction of depressive rumination reduces sleep disturbances and depressive symptoms, whereas inappropriate parenting in childhood has the opposite effect to appropriate parenting. Although it is important for children to receive appropriate parenting in childhood, to prevent the onset of and the exacerbation of depressive symptoms and depression in adulthood,^7^, ^11^, ^12^, ^13^, ^14^ it is not possible to change the parenting they received in the past. As sleep disturbances are also a risk factor for the onset of depression, and are also one of the major symptoms of depression,^9^, ^10^, ^48^ various studies on the prevention and treatment of sleep disturbances have been conducted, and their clinical benefits on depression have also been reported.^49^, ^50^ However, it is imperative that depressed patients with sleep disturbances receive not only preventive care and treatment for sleep disturbances, but also undergo clinical assessment and intervention for depressive rumination, which is closely associated with sleep disturbances. Interventions that take depressive rumination into account are expected to improve sleep disturbances, reduce depressive symptoms, and more effectively prevent the onset and exacerbation of depression. Rumination‐focused cognitive‐behavioral therapy has been developed as a psychotherapy for depressive rumination.^43^ Utilizing it to provide early intervention for depressive rumination is of high clinical significance in the treatment of depression and associated sleep disturbances.

## LIMITATIONS

As the present study was based on a questionnaire to assess the parenting that had been experienced during childhood by subjects aged 20–77 (41.6 ± 12.0) years old, recall bias should be taken into account, particularly in the older adults. However, the validity of the retrospective evaluation by the PBI used in the present study has been confirmed in previous studies.^35^ Adverse childhood experiences (ACEs) have been reported to affect mental and physical health in adulthood.^51^ Numerous retrospective studies on ACEs have been conducted around the world. A prospective study using the Dunedin cohort confirmed the validity of the retrospective assessment of ACEs.^52^ Furthermore, a retrospective study of ACEs in older adults reported that test results were not affected by age or cognitive function.^53^ Therefore, we did not consider recall bias to be a serious problem in the present study. In addition, Table S1 and Figures S1 and S2 indicate that it is possible to discuss pathological depressive symptoms (i.e., MD) based on the results of the present study. However, as the present study was conducted on adult volunteers, there are limitations to whether the findings are applicable to patients with MD. Furthermore, as this is a cross‐sectional study, a long‐term prospective longitudinal study is needed to conclude a causal association among parental attitudes experienced in childhood, depressive rumination, sleep disturbances, and depressive symptoms.

## CONCLUSION

This is the first study to our knowledge to show that depressive rumination fully mediates the association of parental attitudes experienced in childhood with sleep disturbances and depressive symptoms, and that sleep disturbances partially mediate the association between depressive rumination and depressive symptoms. SEM demonstrated associations among the four variables, namely parental attitudes, depressive rumination, sleep disturbances, and depressive symptoms. Based on this study, a large‐scale prospective longitudinal study on community residents is needed in the future to elucidate causal associations among the variables.

## AUTHOR CONTRIBUTIONS

All authors made a significant contribution to the work reported, whether that is in the conception, study design, execution, acquisition of data, analysis and interpretation, or in all these areas; took part in drafting, revising, or critically reviewing the article; gave final approval of the version to be published; have agreed on the journal to which the article has been submitted; and agree to be accountable for all aspects of the work.

## CONFLICT OF INTEREST STATEMENT

The authors have read the journal's policy and the authors of this manuscript have the following competing interests: Yu Tamada has received personal fees from Otsuka Pharmaceutical, Sumitomo Pharma, Eisai, MSD, and Meiji Seika Pharma. Takeshi Inoue has received personal fees from Mochida Pharmaceutical, Takeda Pharmaceutical, Eli Lilly, Janssen Pharmaceutical, MSD, Taisho Toyama Pharmaceutical, Yoshitomiyakuhin, and Daiichi Sankyo; grants from Shionogi, Astellas, Tsumura, and Eisai; and grants and personal compensation from Otsuka Pharmaceutical, Sumitomo Pharma, Mitsubishi Tanabe Pharma, Kyowa Pharmaceutical Industry, Pfizer, Novartis Pharma, and Meiji Seika Pharma; and is a member of the advisory boards of Pfizer, Novartis Pharma, and Mitsubishi Tanabe Pharma. Jiro Masuya has received personal fees from Otsuka Pharmaceutical, Eli Lilly, Astellas, and Meiji Yasuda Mental Health Foundation, and grants from Pfizer. All other authors declare that they have no actual or potential conflicts of interest associated with this study.

## ETHICS APPROVAL STATEMENT

The study protocol was approved by the Ethics Committee of Tokyo Medical University (study approval number: SH3502).

## PATIENT CONSENT STATEMENT

All subjects were informed that participation in this research was voluntary, and the collected information was anonymized so that the individuals could not be identified. Only the subjects who gave their consent to participate in this study were analyzed.

## CLINICAL TRIAL REGISTRATION

Not applicable.

## Supporting information

Supplementary information.

Supplementary information.

## Data Availability

The data used in this study cannot be shared publicly because of Ethics Committee restriction. All relevant data are within the paper. Data are available from the Internal Review Board of the Department of Psychiatry, Tokyo Medical University, Japan (contact via email: seisinka@tokyo-med.ac.jp), for researchers who meet the criteria for access to confidential data.
