# Supplementary material for: Effects of childhood experiences of parental attitude, depressive rumination, and sleep disturbances on adulthood depressive symptoms
Source: PCN Rep. 2024 Jun 24;3(2):e220. doi: 10.1002/pcn5.220 (PMC11196181; doi:10.1002/pcn5.220)
Supplement: Supplementary file 1 — Supplementary information. [file PCN5-3-e220-s001.docx]

**Supporting Information: Supplementary Tables**

**Supplementary Table 1. Results of logistic regression analysis of PHQ-9 score ≥ 10 (major depression) and PHQ-9 score ≤ 4 (no depression) as dependent variables (forced entry method)**

| Independent variable | *p*-value | Odds ratio | 95%CI |
| --- | --- | --- | --- |
| Age | 0.277 | 1.021 | 0.984–1.059 |
| Sex (women) | 0.366 | 1.533 | 0.607–3.872 |
| RRS score | < 0.001 | 1.137 | 1.086–1.191 |
| PBI score  Paternal care  Maternal care  Paternal overprotection  Maternal overprotection |  | | |
|  | 0.731 | 1.013 | 0.942–1.088 |
|  | 0.001 | 0.875 | 0.806–0.950 |
|  | 0.878 | 1.007 | 0.919–1.103 |
|  | 0.462 | 0.969 | 0.890–1.054 |
| PSQI global score | < 0.001 | 1.581 | 1.369–1.826 |

Fit index of this model: χ^2^ = 158.366 (*p* < 0.001); Nagelkerke *R*^2^ = 0.618; Hosmer-Lemeshow test: *p* = 0.800; predictive accuracy = 0.926

The dependent variable was PHQ-9 score ≤ 4 (=1) or PHQ-9 score ≥ 10 (=2). The independent variables were age, sex (women), RRS score, PBI scores, and PSQI global score.

PHQ-9, Patient Health Questionnaire-9; CI, confidence interval; RRS, Ruminative Responses Scale; PBI, Parental Bonding Instrument; PSQI, Pittsburgh Sleep Quality Index

**Supplementary Table 2. Results of multiple regression analysis of total scores of the 8 items of the PHQ-9 without the sleep item (item no. 3) as a dependent variable (forced entry method)**

| Independent variable | Standardized partial regression coefficient (β) | *p*-value | VIF |
| --- | --- | --- | --- |
| Age | –0.007 | 0.860 | 1.397 |
| Sex (women) | 0.054 | 0.151 | 1.142 |
| Marital status (married) | –0.014 | 0.769 | 1.743 |
| Living alone | 0.063 | 0.170 | 1.685 |
| Education (years) | –0.032 | 0.452 | 1.418 |
| Past history of psychiatric disease | 0.110 | 0.010 | 1.436 |
| Current psychiatric disease | –0.018 | 0.660 | 1.386 |
| Family history of psychiatric disease | –0.028 | 0.441 | 1.089 |
| RRS score | 0.338 | < 0.001 | 1.411 |
| PBI score  Paternal care  Maternal care  Paternal overprotection  Maternal overprotection |  | | |
|  | 0.081 | 0.111 | 2.056 |
|  | –0.209 | <0.001 | 2.386 |
|  | 0.106 | 0.046 | 2.252 |
|  | –0.099 | 0.074 | 2.445 |
| PSQI global score | 0.336 | < 0.001 | 1.300 |
| Adjusted *R*^2^ = 0.43; *F* = 25.90; *p* < 0.001 | | | |

VIF, Variance Inflation Factor; RRS, Ruminative Responses Scale; PBI, Parental Bonding Instrument; PSQI, Pittsburgh Sleep Quality Index; PHQ-9, Patient Health Questionnaire-9
