# Supplementary material for: Effects of childhood experiences of parental attitude, depressive rumination, and sleep disturbances on adulthood depressive symptoms
Source: PCN Rep. 2024 Jun 24;3(2):e220. doi: 10.1002/pcn5.220 (PMC11196181; doi:10.1002/pcn5.220)
Supplement: Supplementary file 2 — Supplementary information. [file PCN5-3-e220-s002.docx]

**Supporting Information: Supplementary Figure 1**

**
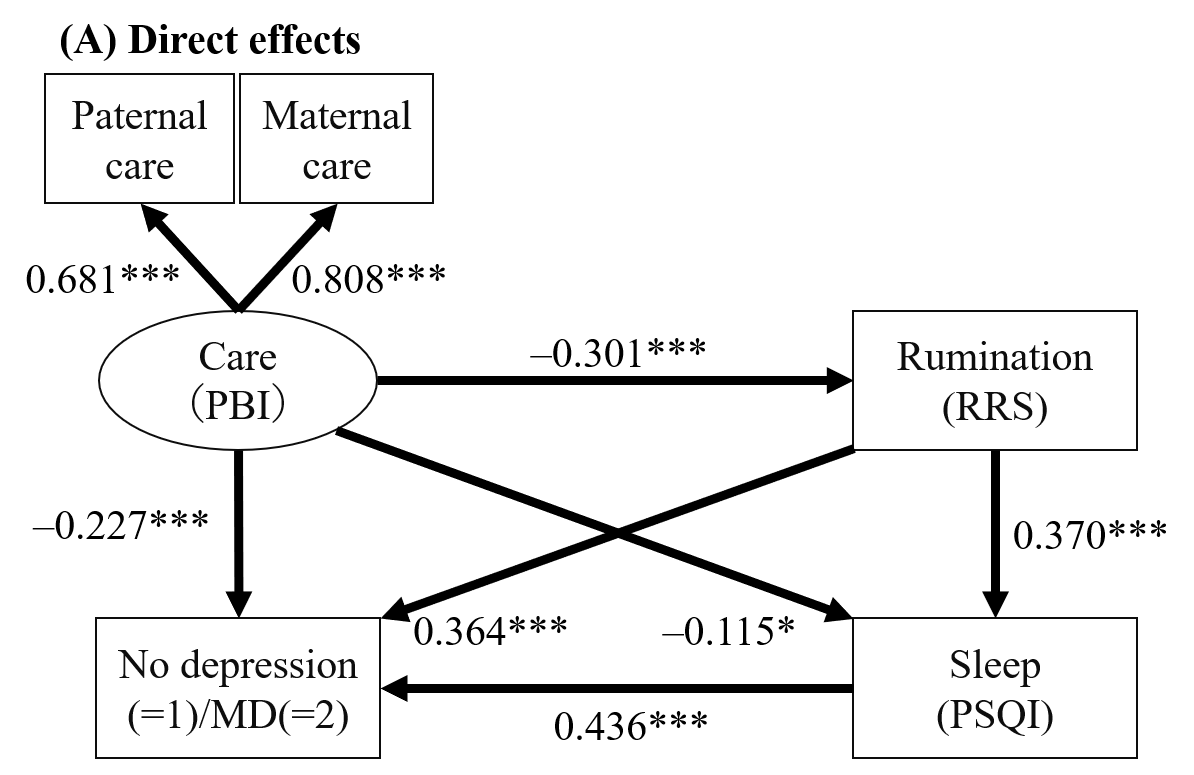
**

**
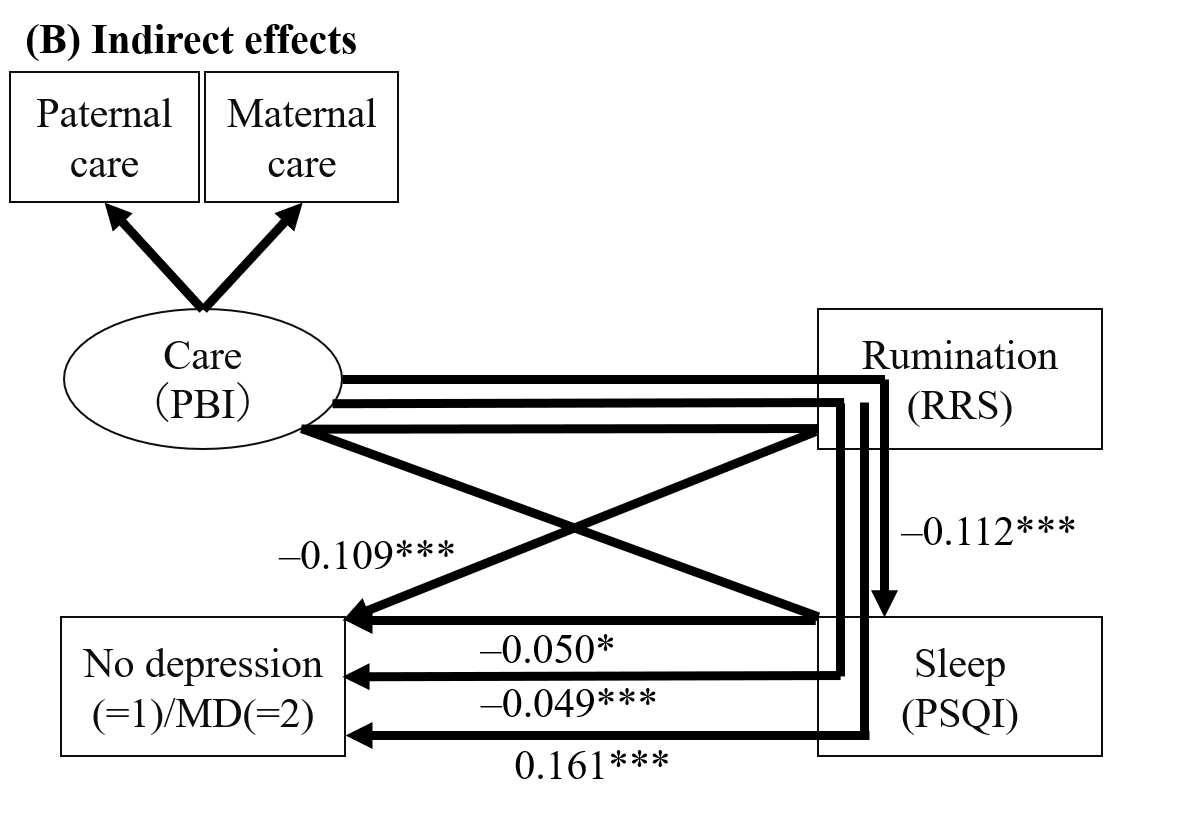
**

**Supplementary Fig. 1. Results of the SEM with the parental attitude of ‘care’ experienced in childhood as the latent variable, and depressive rumination (RRS), sleep disturbance (PSQI), and major depression (MD, PHQ-9 score ≥ 10) or no depression (PHQ-9 score ≤ 4) as the observed variables.** The latent variable is shown as an oval, and the observed variables are shown as rectangles. Direct effects (A) and indirect effects (B) between the variables are shown. The numbers indicate the standardized path coefficients. **p* < 0.05, ****p* < 0.001

**Supplementary Figure 2**

**
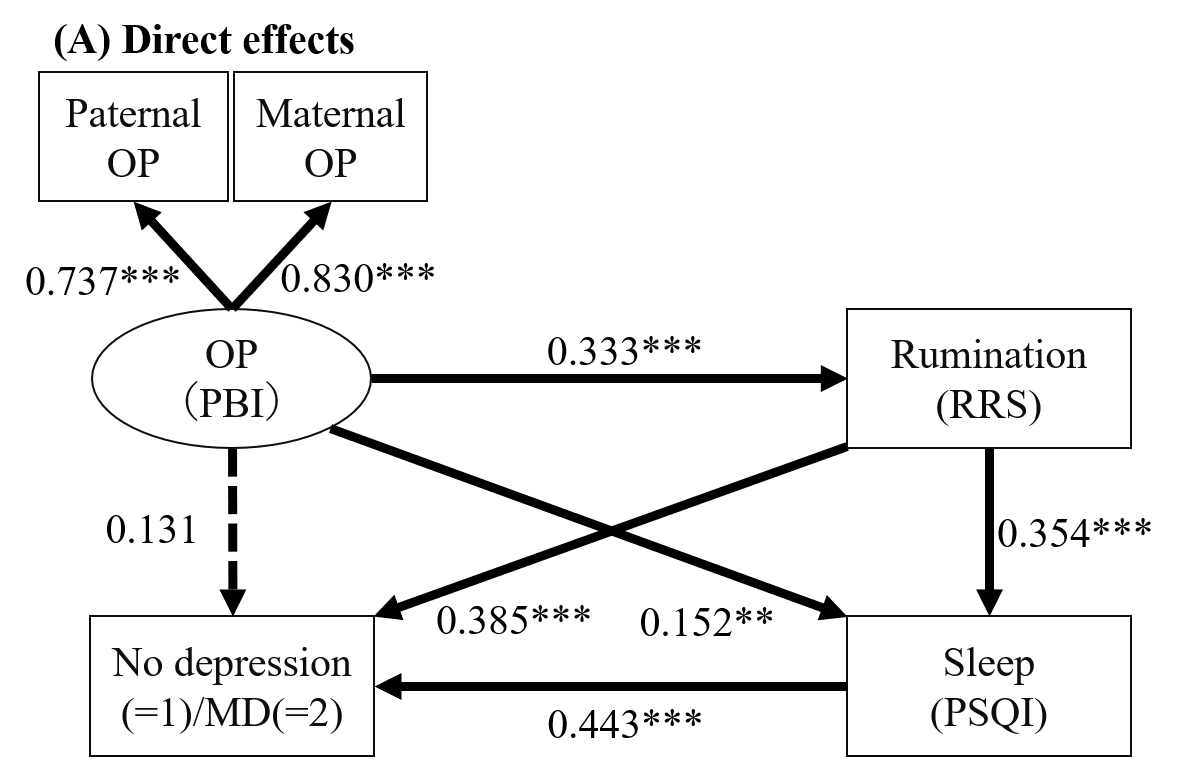
**

**
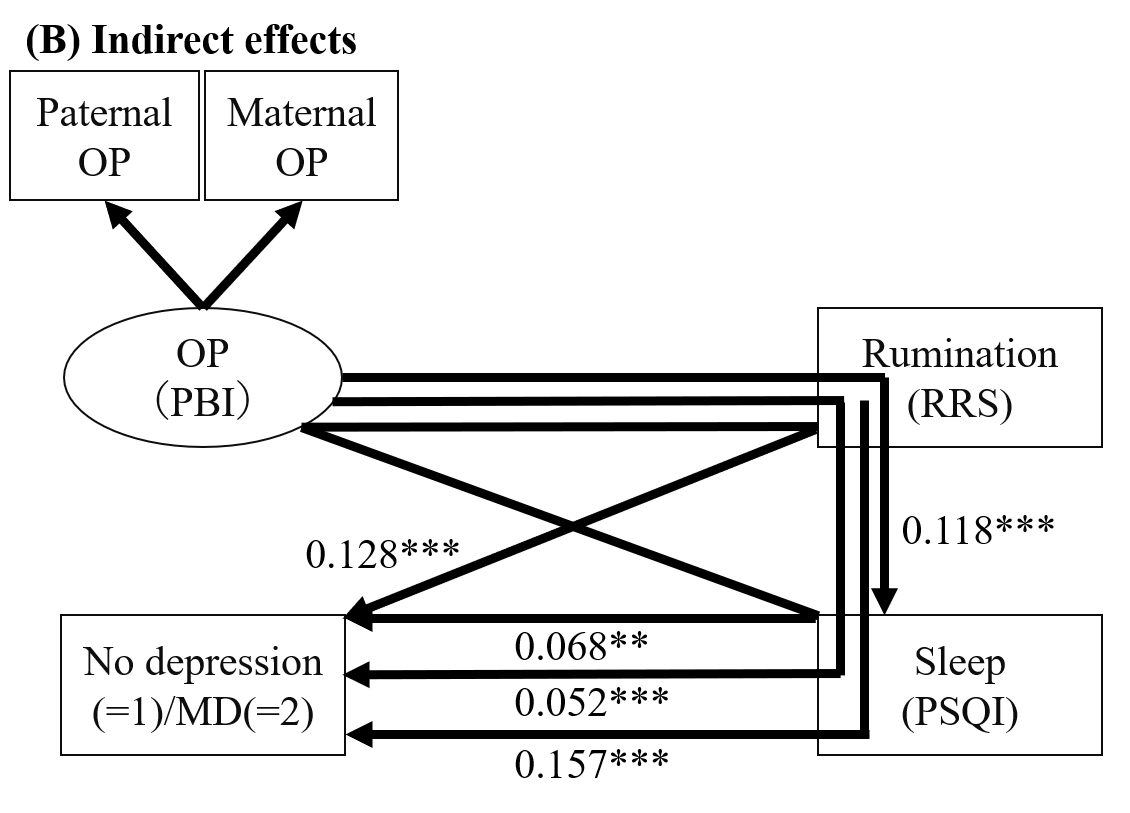
**

**Supplementary Fig. 2. Results of the SEM with the parental attitude of ‘overprotection (OP)’ in childhood as the latent variable, and depressive rumination (RRS), sleep disturbance (PSQI), and major depression (MD, PHQ-9 score ≥ 10) or no depression (PHQ-9 score ≤ 4) as the observed variables.** The latent variable is shown as an oval, and the observed variables are shown as rectangles. The arrows with solid lines represent the statistically significant paths, and those with broken lines represent the nonsignificant paths. Direct effects (A) and indirect effects (B) between the variables are shown. The numbers indicate the standardized path coefficients. ***p* < 0.01, ****p* < 0.001

**Supplementary Figure 3**


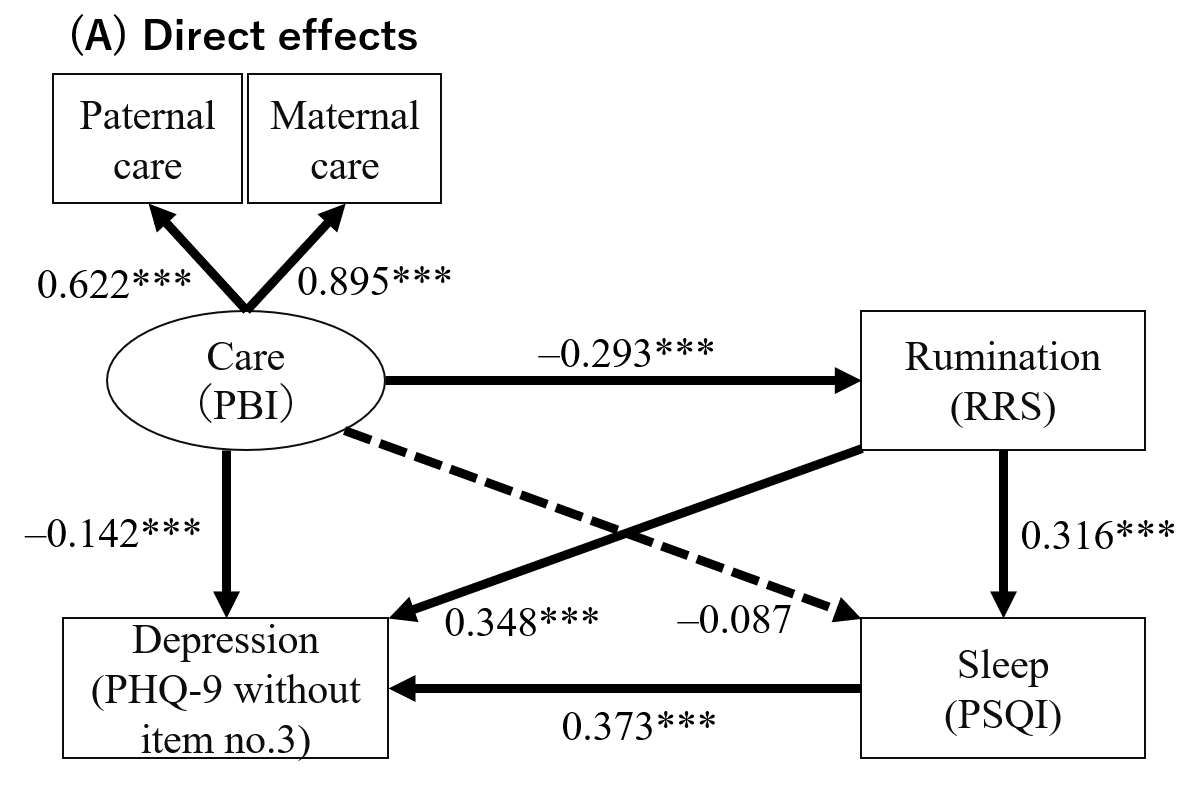


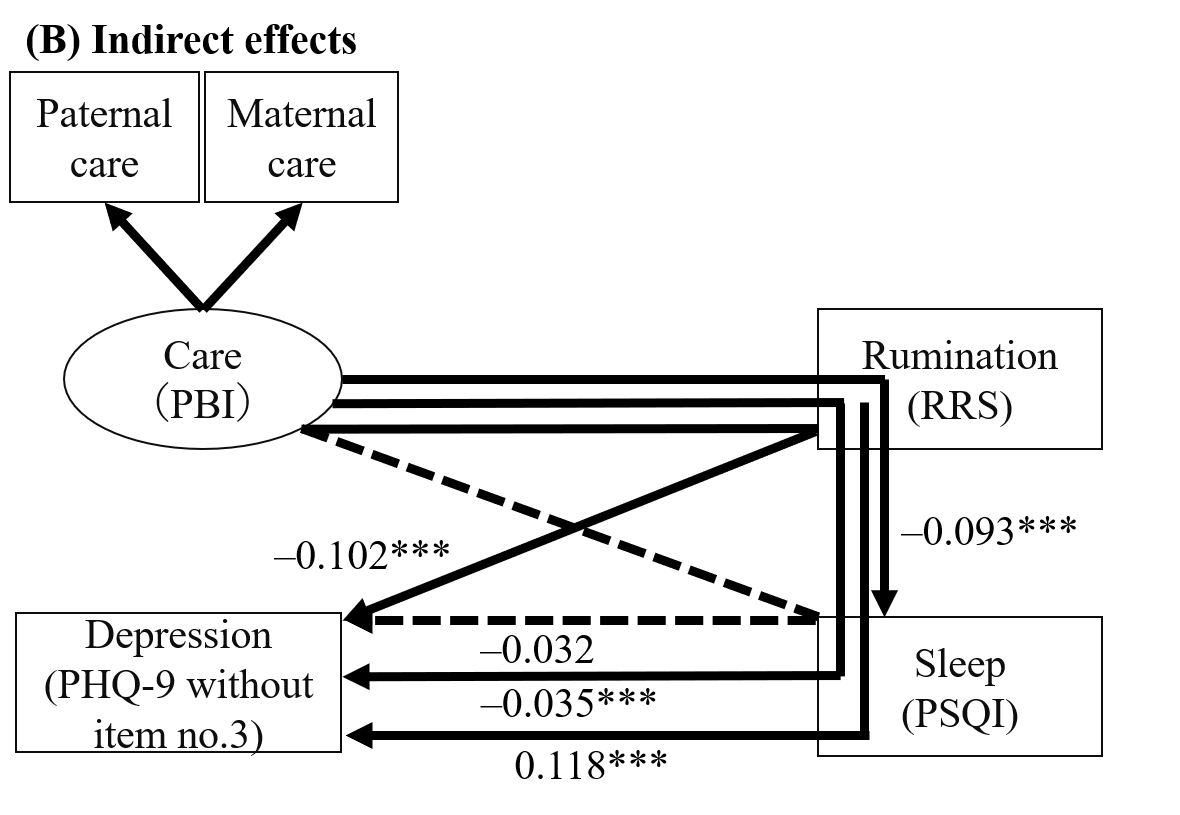


**Supplementary Fig. 3. Results of the SEM with the parental attitude of ‘care’ experienced in childhood as the latent variable, and depressive rumination (RRS), sleep disturbance (PSQI), and total score of the 8 items of PHQ-9 without the score of the sleep item (item no. 3) as the observed variables.** The latent variable is shown as an oval, and the observed variables are shown as rectangles. The arrows with solid lines represent the statistically significant paths, and those with broken lines represent the nonsignificant paths. Direct effects (A) and indirect effects (B) between the variables are shown. The numbers show the standardized path coefficients. ****p* < 0.001

**Supplementary Figure 4**

**
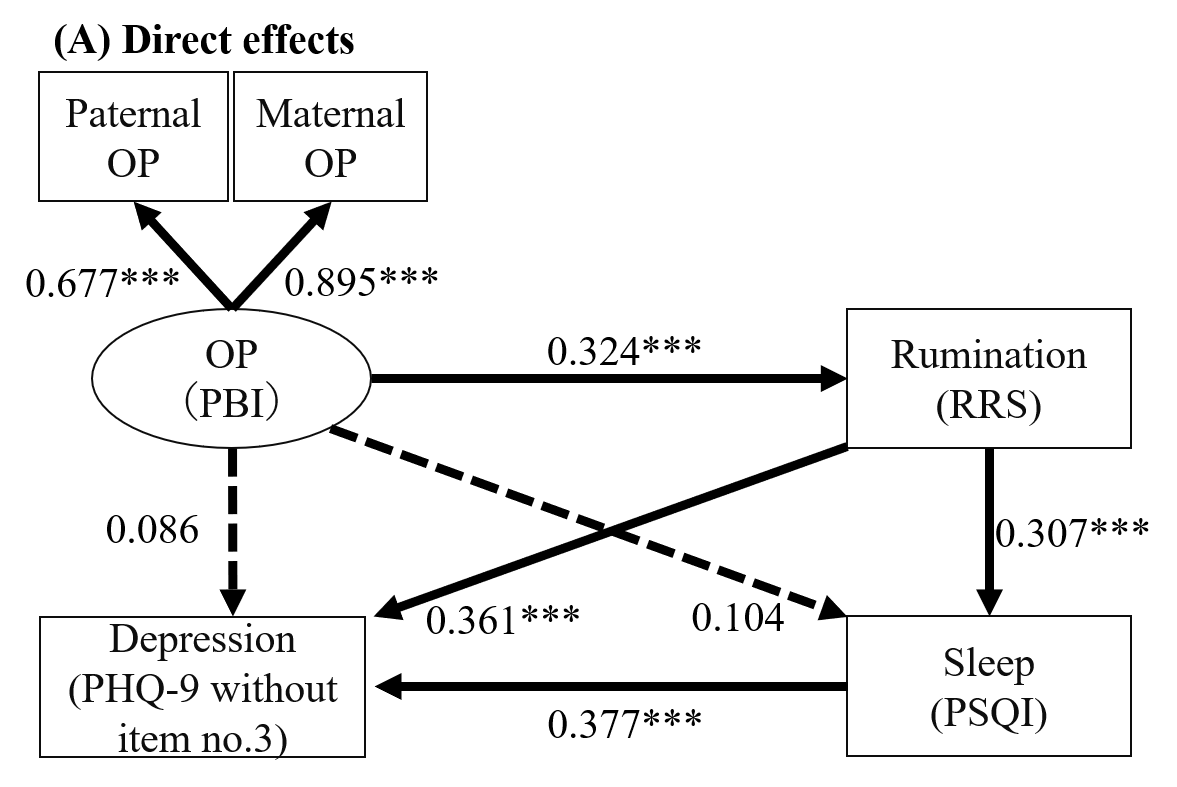
**

**
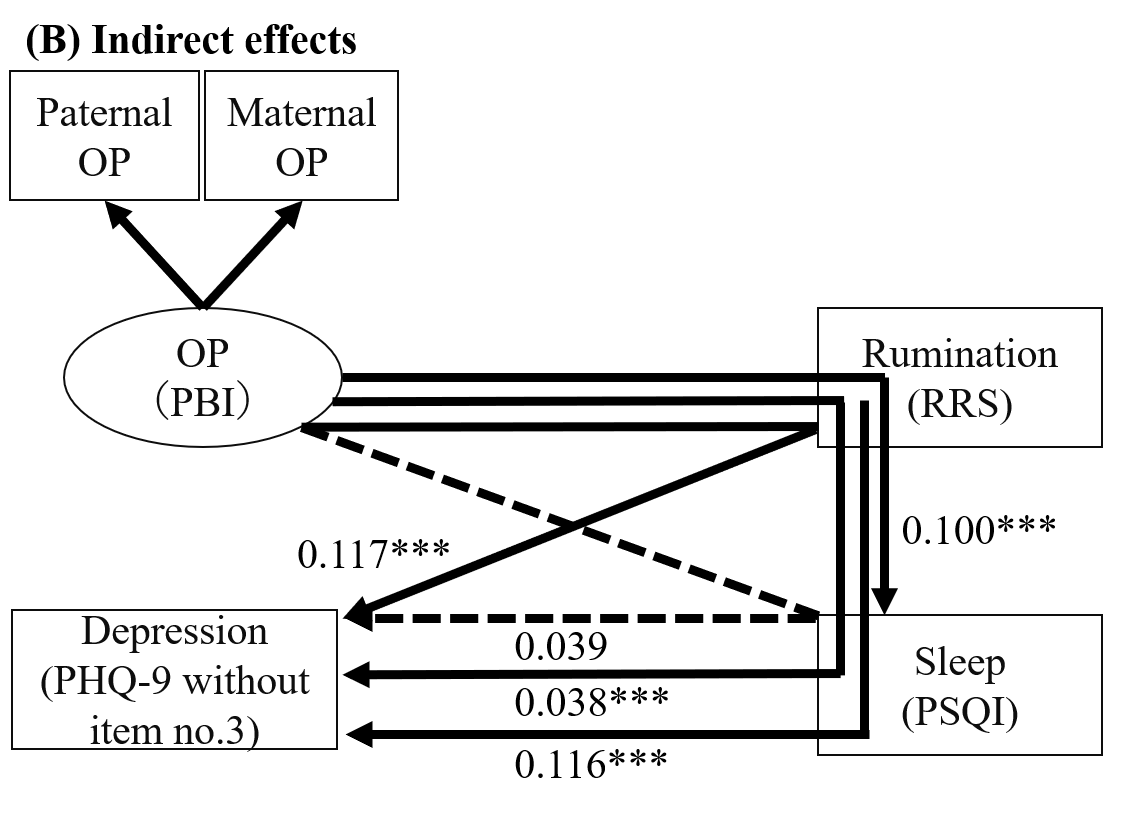
**

**Supplementary Fig. 4. Results of the SEM with the parental attitude of ‘overprotection (OP)’ in childhood as the latent variable, and depressive rumination (RRS), sleep disturbance (PSQI), and total score of the 8 items of PHQ-9 without the score of the sleep item (item no. 3) as the observed variables.** The latent variable is shown as an oval, and the observed variables are shown as rectangles. The arrows with solid lines represent the statistically significant paths, and those with broken lines represent the nonsignificant paths. Direct effects (A) and indirect effects (B) between the variables are shown. The numbers show the standardized path coefficients. ****p* < 0.001

**Supplementary Figure 5**

**
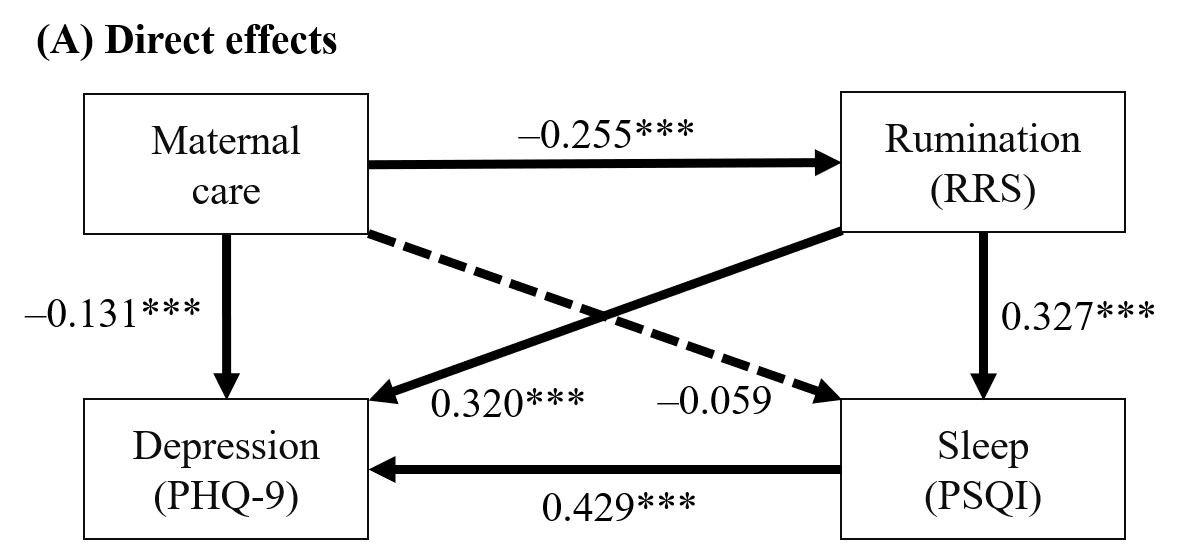
**

**
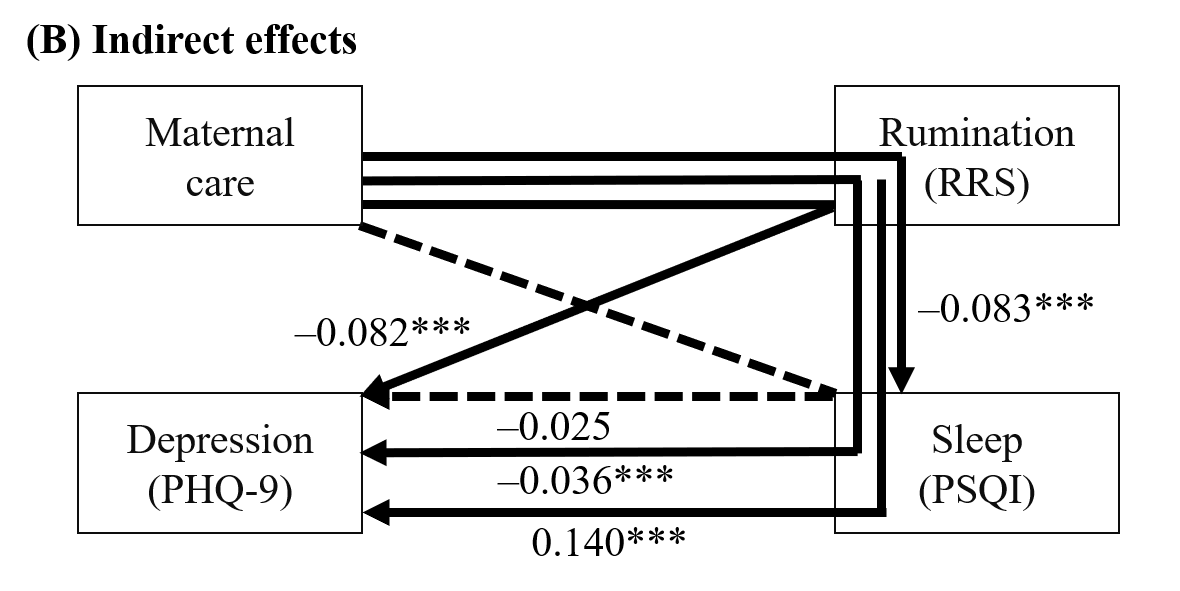
**

**Supplementary Fig. 5. Results of the path analysis with the parental attitude of ‘maternal care’ experienced in childhood, depressive rumination (RRS), sleep disturbance (PSQI), and depression evaluated on the PHQ-9 as the observed variables.** The observed variables are shown as rectangles. The arrows with solid lines represent the statistically significant paths, and those with broken lines represent the nonsignificant paths. Direct effects (A) and indirect effects (B) between the variables are shown. The numbers show the standardized path coefficients. ****p* < 0.00

**Supplementary Figure 6**

**
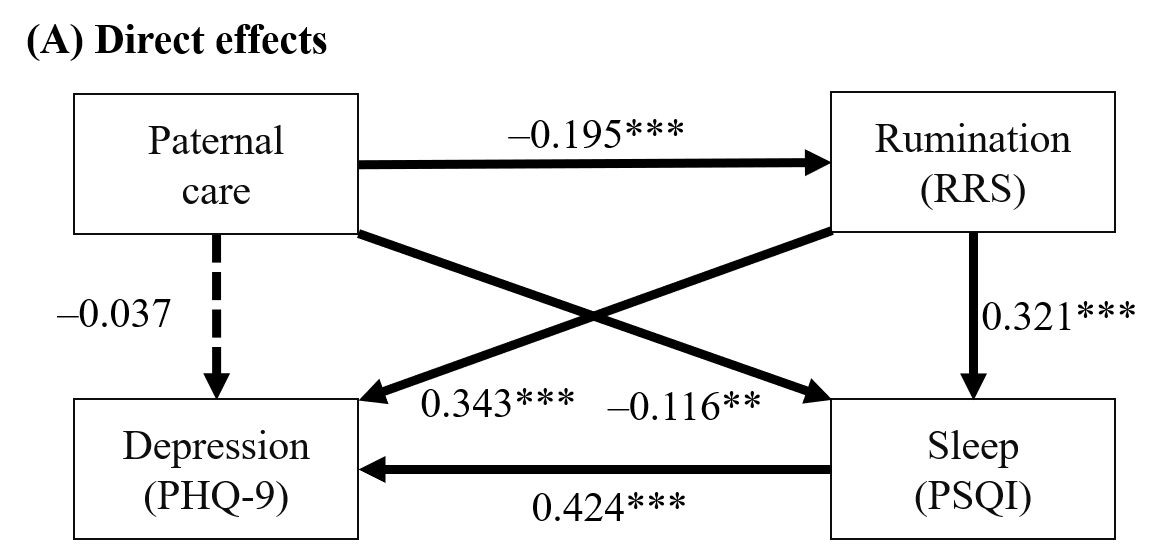
**

**
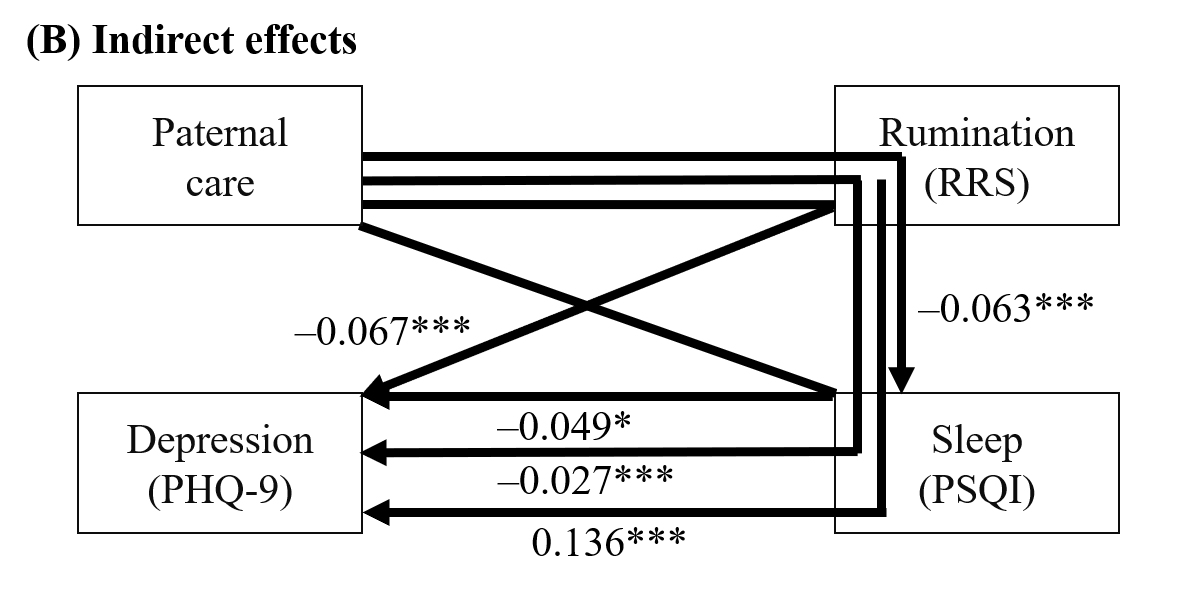
**

**Supplementary Fig. 6. Results of the path analysis with the parental attitude of ‘paternal care’ experienced in childhood, depressive rumination (RRS), sleep disturbance (PSQI), and depression evaluated on the PHQ-9 as the observed variables.** The observed variables are shown as rectangles. The arrows with solid lines represent the statistically significant paths, and those with broken lines represent the nonsignificant paths. Direct effects (A) and indirect effects (B) between the variables are shown. The numbers show the standardized path coefficients. **p* < 0.05, ***p* < 0.01, ****p* < 0.001
